# RNA sequencing identifies key genes involved in intramuscular fat deposition in chickens at different developmental stages

**DOI:** 10.1186/s12864-023-09819-y

**Published:** 2024-02-27

**Authors:** Jinmei Zhu, Yongli Wang, Yongchun Su, Maiqing Zheng, Huanxian Cui, Zhiwu Chen

**Affiliations:** 1 College of Animal Science and Technology, Qingdao Agricultural University, Qingdao, 266109 China; 2 State Key Laboratory of Animal Nutrition, Key Laboratory of Animal (Poultry) Genetics Breeding and Reproduction, Ministry of Agriculture, Institute of Animal Sciences, Chinese Academy of Agricultural Sciences, Beijing, 100193 China; 3 Guangxi Jingling Agriculture and animal Husbandry Group Co., LTD, Nanning, 530049 China

**Keywords:** Intramuscular fat, RNA sequencing, WGCNA, Hub gene, Meat quality

## Abstract

**Background:**

Intramuscular fat (IMF) is an important factor in meat quality, and triglyceride (TG) and Phospholipids (PLIP), as the main components of IMF, are of great significance to the improvement of meat quality.

**Results:**

In this study, we used 30 RNA sequences generated from the transcriptome of chicken breast muscle tissues at different developmental stages to construct a gene expression matrix to map RNA sequence reads to the chicken genome and identify the transcript of origin. We used weighted gene co-expression network analysis (WGCNA) and identified 27 co-expression modules, 10 of which were related to TG and PLIP. We identified 150 highly-connected hub genes related to TG and PLIP, respectively, which were found to be mainly enriched in the adipocytokine signaling pathway, MAPK signaling pathway, mTOR signaling pathway, FoxO signaling pathway, and TGF-beta signaling pathway. Additionally, using the BioMart database, we identified 134 and 145 candidate genes related to fat development in the TG-related module and PLIP-related module, respectively. Among them, *RPS6KB1, BRCA1, CDK1, RPS3, PPARGC1A, ACSL1, NDUFAB1, NDUFA9, ATP5B* and *PRKAG2* were identified as candidate genes related to fat development and highly-connected hub genes in the module, suggesting that these ten genes may be important candidate genes affecting IMF deposition.

**Conclusions:**

*RPS6KB1, BRCA1, CDK1, RPS3, PPARGC1A, ACSL1, NDUFAB1, NDUFA9, ATP5B* and *PRKAG2* may be important candidate genes affecting IMF deposition. The purpose of this study was to identify the co-expressed gene modules related to chicken IMF deposition using WGCNA and determine key genes related to IMF deposition, so as to lay a foundation for further research on the molecular regulation mechanism underlying chicken fat deposition.

## Introduction

With the continuous development of the broiler industry, the consumption of chicken as the main source of animal protein in China is increasing every year [1]. However, high-density breeding and high-intensity growth selection lead to continuous decline in the quality of chicken, due to changes in lipids and aroma compounds in chicken intramuscular fat (IMF), IMF are important precursors of flavor directly affecting the tenderness, flavor, color and juiciness of chicken meat, and thus are closely related to meat quality [2]. Therefore, to improve meat quality it is extremely important to elucidate the molecular mechanism underlying IMF deposition.

The mechanism of IMF deposition is a very complex, dynamic process consisting of a series of steps, such as adipocyte differentiation, lipid synthesis, transport and decomposition [3], which is affected by age, nutrition, environment and other factors [4]. It involves many metabolic pathways and related genes, such as *PPARG, GPAT1, FABP4* and *FABP5* [5], as well as signal pathways, such as IGF/IGF-1, MAPK, TGF-β /BMPs, Wnt/β-catenin [6], and gene expression varies at different stages. The number and volume of adipocytes are closely related to the content of IMF. Fat in other animals such as pigs is mainly synthesized in adipose tissue, while fat in poultry such as chicken is mainly synthesized in the liver [7]. Most chicken fatty acids are first synthesized in the liver and then transported by low-density lipoproteins or chylomicrons for storage in adipose tissue as triglycerides (TG), while exogenous fat is absorbed through the small intestine and transported to various target tissues [8], such as fat and muscle tissues, in the form of chylous particles. These fats are eventually hydrolyzed into fatty acids by lipoproteases and then oxidized to provide energy or deposit [9] in the target tissue.

The main components of IMF are TG, phospholipids (PLIP) and cholesterol [10]. TG are composed of glycerol and fatty acids, and as the main component of IMF play a crucial role in muscle lipid metabolism [11]. PLIP are a major component of cellular membranes and IMFs, which are hydrolyzed to produce a mixture containing fatty acids and phosphoric acid [12], and due to their rich fatty acid content PL are the main flavor compound, which is closely related to meat quality. Therefore, it is critical to study the role of TG and PLIP in the process of IMF deposition.

With the development of second-generation sequencing, transcriptome sequencing has been widely used in the study of gene expression and regulation at the genome-wide level [13]. There are a growing number of studies on chicken IMF deposition using transcriptome sequencing technology. Numerous genes involved in IMF and abdominal fat deposition in broilers have been identified through transcriptome data differential expression analysis and weighted gene co-expression network analysis (WGCNA) [14]. To date, many genes have been confirmed to be related to chicken IMF deposition. Differential expression analysis and WGCNA are currently the main methods available for analyzing transcriptome data [15]. Differential expression analysis is mainly used to analyze the differences in expression between samples from two groups and WGCNA is a method to construct a gene co-expression network based on gene expression data [16], which mainly analyzes the relationship between the co-expression of genes. To analyze the relationship between the co-expression of genes, genes are separated into multiple modules, and then key genes related to phenotypes are identified through correlation analysis between modules and sample phenotypes [17]. Therefore, this study mainly constructed a co-expression network based on the transcriptome data of pectoral muscle tissues at six different developmental stages using the WGCNA method to study the regulation of IMF deposition and identified key genes related to the regulation of IF deposition. In addition, this study provides a more comprehensive theoretical basis for the molecular mechanism underlying IMF deposition.

## Materials and methods

### Experimental material

The Jinxing yellow (JXY) chickens obtained from the Institute of Animal Sciences of Chinese Academy of Agricultural Sciences (Beijing, China) of different ages used in this study were fed the same diet and raised under the same conditions at the Changping Experimental Base of the Institute of Animal Sciences of Chinese Academy of Agricultural Sciences (Beijing, China). The chickens were stunned by electric shock and killed by cervical dislocation at different developmental stages. Samples were collected at six different developmental stages: D1 (the first day after birth), D7, D35, D63, D91, and D119. Body weight, breast muscle weight, and abdominal fat weight were recorded at slaughter, and tissue samples of breast muscle were collected, quickly frozen in liquid nitrogen, and stored at -80 °C. The specific sample information is shown in Table 1.

**Table 1:** Sample information

Stage	Breed	Number of samples	Tissue
D1	JXY	7	pectoral muscle
D7	JXY	4	pectoral muscle
D35	JXY	5	pectoral muscle
D63	JXY	5	pectoral muscle
D91	JXY	4	pectoral muscle
D119	JXY	5	pectoral muscle

### Collection of phenotypic data related to intramuscular fat

In order to obtain phenotypic traits related to IMF, the contents of TG and PLIP were determined in pectoral muscle samples at six different developmental stages: D1 (the first day after birth), D7, D35, D63, D91, and D119. Briefly, samples of 2.0 g of breast muscle tissue of JXY chicken were homogenized and analyzed to determine the content of TG and PL in breast muscle tissue [18], using the appropriate detection kits (Nanjing Jiancheng Bioengineering Research Institute of China and Beijing Leichuang Biotechnology Co., Ltd. of China).

### RNA library construction and transcriptome sequencing analysis

Transcriptome sequencing was performed by Beijing Guoke Biological Technology Co., Ltd. (Beijing, China). Total RNA was extracted using TRIZOL reagent (Invitrogen, Carlsbad, CA, USA). To ensure the accuracy of the data, before RNA sequencing (RNA-seq) we determined the purity (OD260/280), concentration, and nucleic acid absorption peak of the RNA using a Nanodrop spectrophotometer (Thermo Fisher Scientific Inc., Waltham, MA, USA), and the RNA integrity using an Agilent 2100 Bioanalyzer system (Agilent Technologies Inc., Santa Clara, CA, USA). After determining that the sample met the RNA quality criteria (based on purity and RNA integrity index), the library was constructed, and the library quantity and quality were determined using Qubit 2.0 for preliminary quantification. In addition, before proceeding to the subsequent experiment, the insert size of the library was detected using the Agilent 2100 Bioanalyzer system and determined to meet the expectations and library inspection was completed by accurately quantifying the effective concentration of the library (library effective concentration > 2nM) by quantitative real-time polymerase chain reaction (qPCR). After determining that the quantity and quality requirements of the library were met, different libraries were pooled according to the target amount of data of the sequencing machine, and sequenced using the Illumina Novaseq platform (Illumina Inc., San Diego, CA, USA). High-quality sequencing sequencing data (clean reads) were obtained by removing reads containing adapter, reads containing ploy-N and low quality reads from raw data. In addition, Q20, Q30, GC-content and sequence duplication level of the clean data were determined. All the downstream analyses were based on high-quality clean data [19]. Raw sequences were transformed into clean reads after data processing, and the clean reads were then mapped to the chicken reference genome sequence. Only reads with a perfect match or one mismatch were further analyzed and annotated based on the reference genome [20].

### Construction of the weighted co-expression network

Using the Illumina sequencing platform to obtain transcriptome data, 21,987 genes were identified from the breast muscle samples of JXY chicken at six different developmental stages, namely D1 (the first day after birth), D7, D35, D63, D91, and D119. The data were used to construct the co-expression network by WGCNA using the WGCNA package in the R software. Abnormal samples in WGCNA will affect the analysis of module results, so before WGCNA, abnormal outlier samples were removed, 30 samples were clustered, and no outlier samples were found. The 21,987 genes identified were used to build a co-expression module, by first calculating the correlation coefficient of genes. In order to determine whether genes have similar expression patterns, it is necessary to set an appropriate soft threshold [21], so that the connection between genes in the network obeys the scale-free network. When the scale-free fitting index is 0.9, the soft threshold is 4(Fig. 2B), indicating good network connectivity. Therefore, 4 is chosen as the most appropriate soft threshold for building a co-expression module. Then, a co-expression network [22] was built through an appropriate soft threshold, and genes were classified according to their expression pattern. Genes with similar expression pattern were classified into one module, and different colors represent different modules. To associate modules with traits, principal component analysis [23] was performed on all genes within the modules. The traits related to IMF, mainly included TG and PLIP. The correlation of modules with IMF-related traits was calculated and displayed as a heatmap. At p < 0.05, modules were considered to be significantly associated with phenotypic traits.

### Screening of hub genes

In WGCNA, gene significance (GS) refers to the absolute value of the correlation between genes and phenotypic traits, and module membership (MM) to the correlation of the module eigengene and the gene expression profile. Genes that are highly correlated with a phenotypic trait are important genes in the modules associated with that trait [22]. Thus, we selected the gene with high GS and MM values as the hub gene in the module and set |GS|>0.2, and the gene at |MM|>0.8 is the hub gene in each corresponding module. We then used Cystoscope 3.9.0 to construct the gene co-expression network to identify the top 30 genes with the highest degree of connectivity in the module as true hub genes and visualize them [24].

### Functional enrichment analysis of modules and selection of candidate genes

Gene ontology (GO; http://www.geneontology.org/) is widely used in bioinformatics research, and the gene product aspects are divided into three categories: biological process (BP), cellular component (CC), and molecular function (MF). The Kyoto Encyclopedia of Genes and Genomes (KEGG, http://www.genome.jp/kegg/) pathway enrichment analysis can identify the metabolic and signal transduction pathways in which the proteins encoded by regulated genes are involved. In order to investigate the key modules of the hub genes, the KOBAS (version 3.0) software (http://kobas.cbi.pku.edu.cn/kobas3/?t=1) was used to identify enriched KEGG pathways and GO terms associated with significant phenotypic traits (P < 0.05 was considered significant). The genes in the module were annotated using the BioMart database (http://www.biomarbiomart.org/) [25] with the reference genome GRCg6a. We annotated the results based on their function and selected genes associated with fat development. Based on the above data, candidate genes affecting fat deposition and development were identified.

### Quantitative real-time PCR (qPCR)

In order to verify the accuracy of RNA-seq analyses, three genes were selected for qPCR verification, primers, based on the sequence of each gene, were designed by the NCBI/Primer-Blast software, and sequence alignment was performed by BLASTN. Total RNA was extracted from each pectoral muscle tissue using TRIzol reagent (Invitrogen) and reverse transcribed using (2.0 µg) of total RNA each sample using the reverse transcription kit FastQuant RT Kit (Tiangen, Beijing, China) to obtain the cDNA. The qPCR reaction was performed in final volume of 10 µL, using 5 µL of 2 × iQTM SYBR Green Supermix, 0.5 µL of upstream primer, 0.5 µL of downstream primer, and 3 µL of cDNA. The samples were subjected to 40 cycles of amplification (95 °C 3 min, 95 °C 3 s, 60 °C 34 s) on a QuantStudio 7 Flex system (Applied Biosystems, Foster City, CA, USA). Amplification of each sample was performed in triplicate. ACTB was used as the internal reference gene, and the collected data were analyzed by the 2^−ΔΔCT^ method.

### Statistical analyses

The significance of differences between means was assessed using the IBM SPSS Statistics (version 26) software (IBM Corporation, Armonk, NY, USA). A *P*-value of less than 0.05 was considered statistically significant. Data are presented as the mean ± standard error of the mean.

## Results

### Phenotypic data associated with intramuscular fat

We measured the contents of TG and PLIP in the breast muscle tissue of JXY chickens at different developmental stages (Table 2). With increasing age, the contents of TG and PLIP increased, and the PLIP and TG content at ages 35 days and above was significantly higher than that at 1 and 7 days old. Also, there was no significant difference between the contents of TG at 1 and 7 days of age.

**Table 2:** Sample phenotypic information

Items	D1	D7	D35	D63	D91	D119
TG	0.56 ± 0.17^c^	0.41 ± 0.1^c^	2.68 ± 0.86^b^	4.58 ± 0.73^a^	4.34 ± 1.01^a^	4.59 ± 1.5^a^
PLIP	2.13 ± 0.34^e^	2.54 ± 0.12^d^	3.71 ± 0.36^b^	3.49 ± 0.24^bc^	3.3 ± 0.22^c^	4.14 ± 0.21^a^

Data were represented by mean ± SEM, and a, b, c, d and e, indicate statistically significant markers. Data with different superscript letters showed significant difference (*P* < 0.05)

TG, triglycerides; PLIP, phospholipids

### Quantitative real-time PCR (qPCR)

**Fig. 1:**
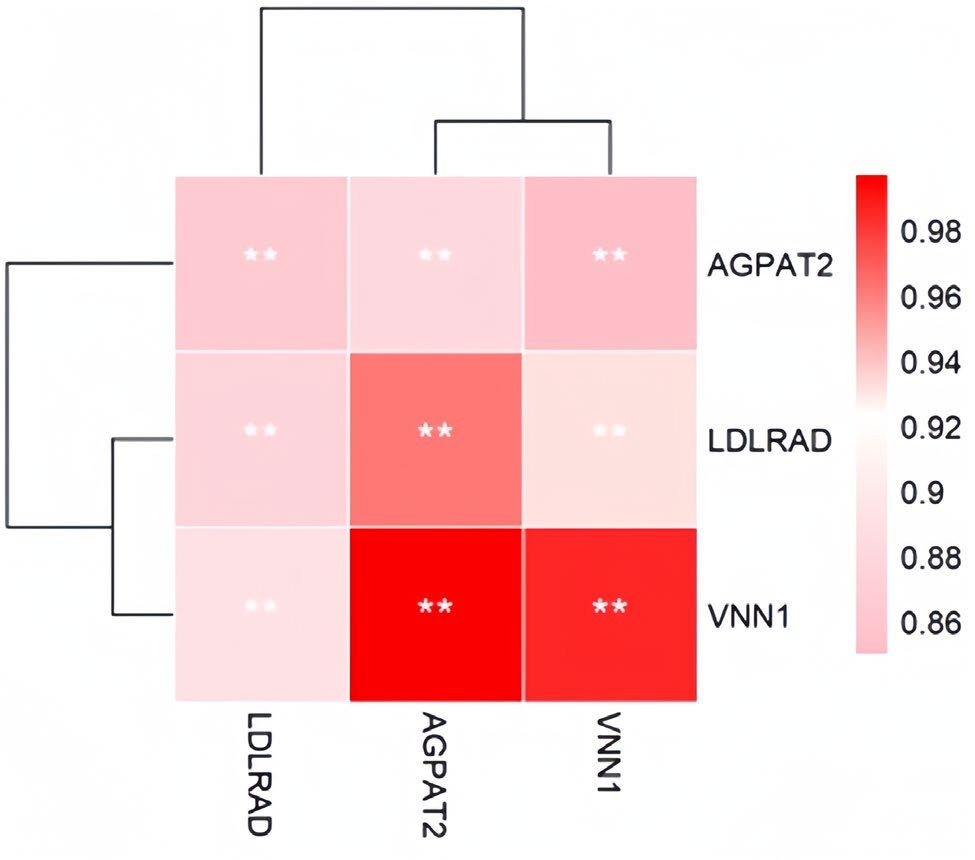
Correlation analysis of qPCR and RNA-seq data, the x-axis is the transcriptome expression value of the gene, and the y-axis is the qPCR value of the gene. * Indicates *P* < 0.05; ** indicates *P* < 0.01

The fluorescence qPCR analysis of three selected genes showed that their expression trend was consistent with the transcriptome sequencing results. The qPCR analysis results were significantly correlated with those of the transcriptome sequencing analysis results, indicating that the transcriptome sequencing data were reliable as Fig. 1.

### Construction of co-expression modules

The gene expression matrix was constructed using the RNA sequences from chicken breast muscle tissues at different ages. Analysis of the 30 samples using the flashClust toolkit (R language), revealed that there were no outlier samples, so all samples were retained. The clustering diagram of the 30 samples is shown in Fig. 2A. Based on the scale-free network, the most suitable soft threshold was chosen to construct the co-expression network. When the scale-free fitting index is 0.9, the soft threshold is 4 (Fig. 2B), so 4 was chosen as the most suitable soft threshold to construct the co-expression module. The genes were divided into different modules, and the gene clustering tree was drawn. The mergeCutHeight for modules generation was set to 0.2, merge modules with a height lower than 0.2 and the minimum number of genes in a module was set to 100 to obtain 27 co-expression modules (Fig. 2C), and the number of genes in these modules ranged from 106 to 9765 (Table 3).

**Fig. 2A:**
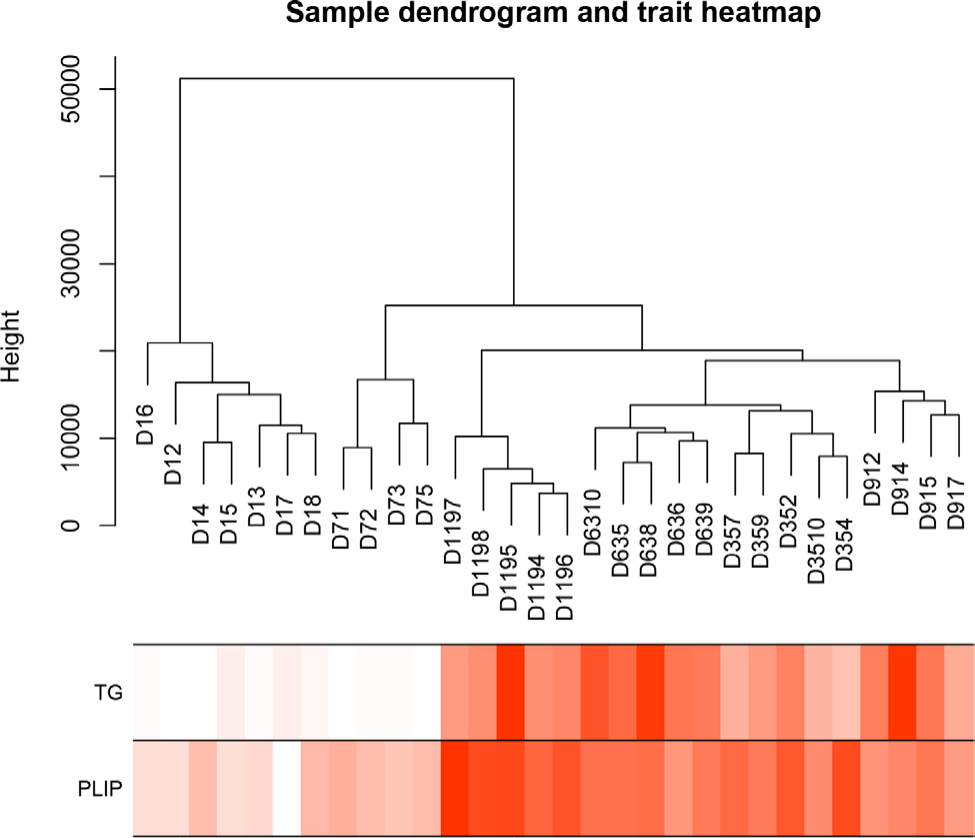
Sample clustering. There are no abnormal samples in the clustering tree

**Fig. 2B:**
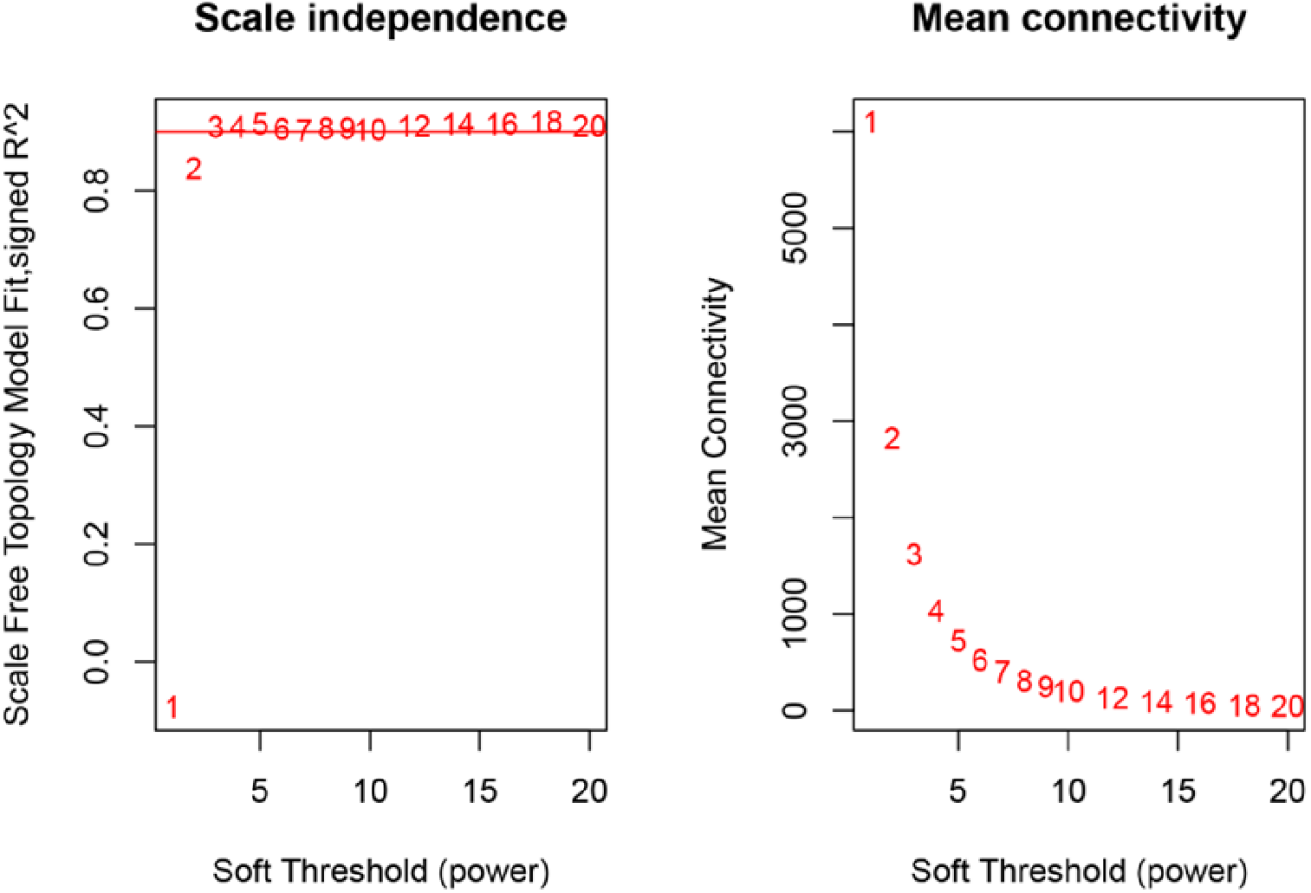
R2 = 0.9, β = 5; when R2 = 0.9, the optimal soft threshold is 5

**Fig. 2C:**
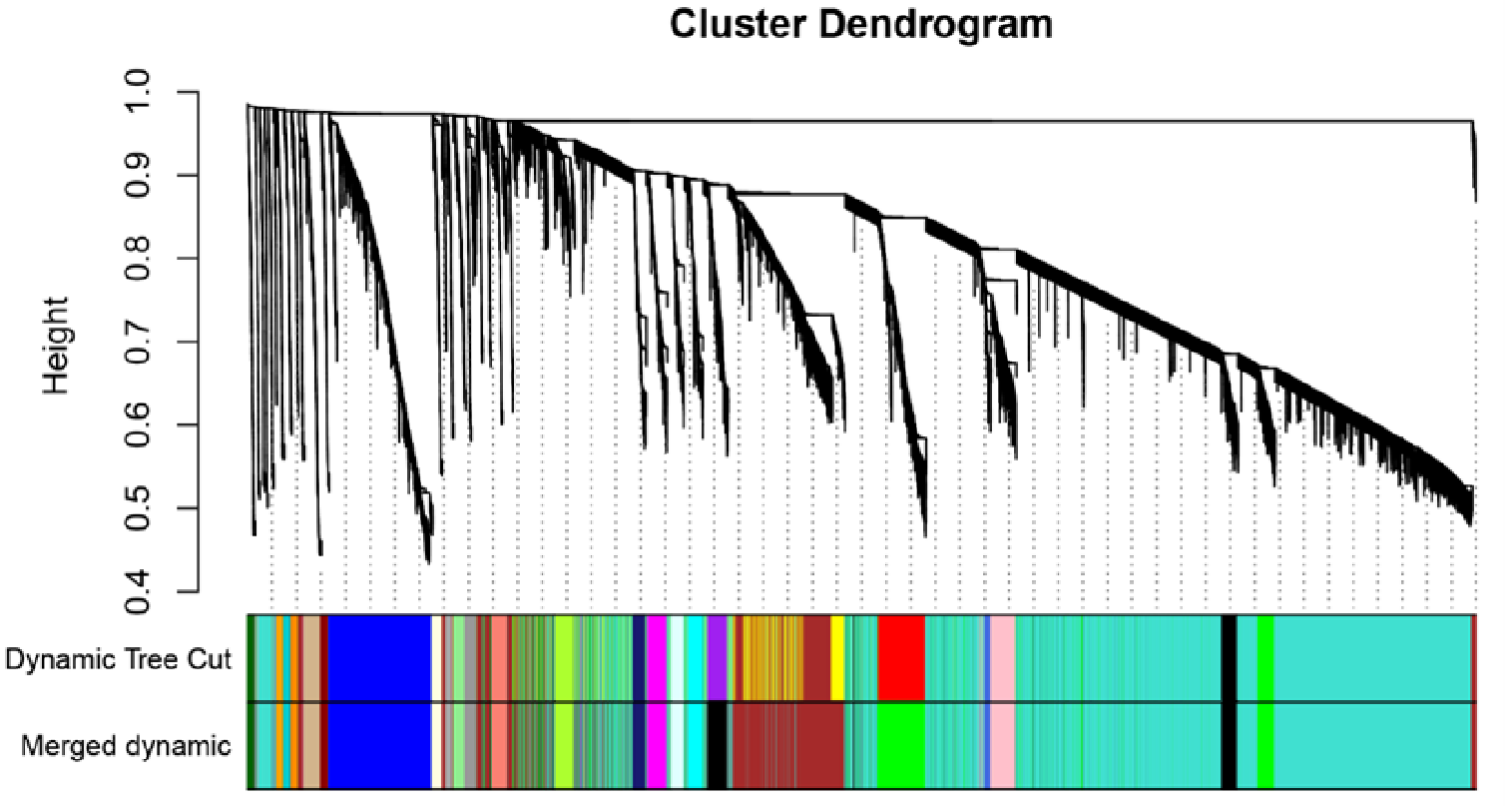
Gene clustering tree. The upper part of the figure is the gene hierarchy clustering tree, and the lower part is the gene module. Each color represents a module

**Table 3:** The number of genes in the 21 modules

Module	Number of genes	Module	Number of genes
ME black	575	ME light yellow	165
ME blue	1750	ME magenta	341
ME brown	1706	ME midnight blue	222
ME cyan	244	ME pink	544
ME dark red	133	ME red	800
ME green	880	ME royal blue	144
ME green-yellow	323	ME salmon	254
ME dark orange	106	ME tan	291
ME grey 60	192	ME turquoise	9765
ME dark grey	116	ME dark green	123
ME light cyan	208	ME yellow	1251
dark turquoise	120	ME orange	111
ME light green	186	purple	336
ME grey	17		

ME, module eigengene

### Module-phenotype correlation analysis

**Fig. 3:**
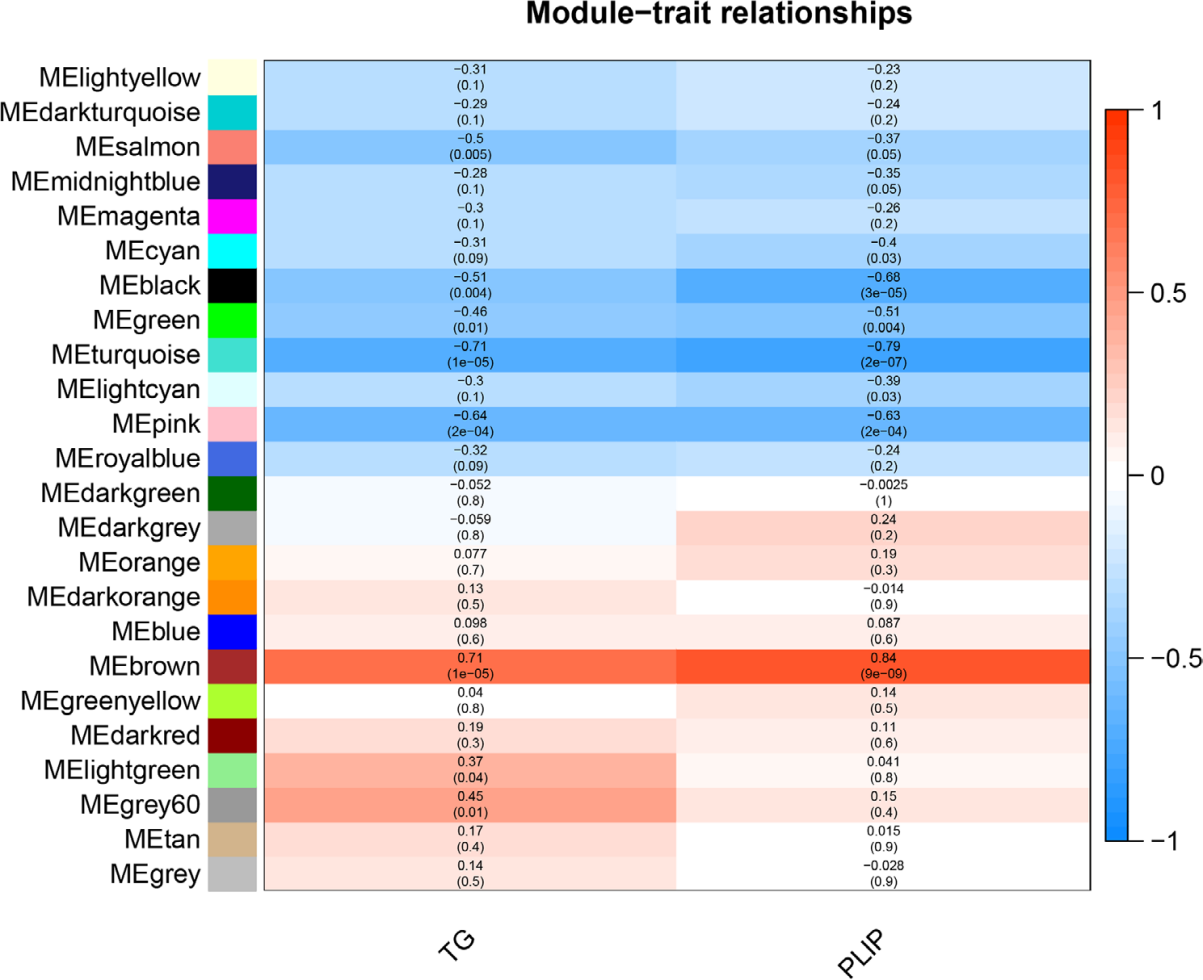
Correlation heat map between phenotype and module, The X-axis represents phenotypic information, and the Y-axis represents modules. The darker the color, the higher the correlation. Red represents positive correlation, blue represents negative correlation, and P < 0.05 indicates a significant correlation

Phenotypic traits associated with IMF include TG and PLIP contents. A module eigengene (ME) refers to the level of gene expression in the module, and the phenotype-module-related heat map is shown in Fig. 3. A total of 10 modules were found to be significantly associated with the IMF trait of the pectoral muscle (P < 0.05). Eight modules, namely the brown, pink, turquoise, green, black, salmon, light green and grey60, were significantly correlated with TG content (P < 0.05), among which the salmon, black, pink, green, and turquoise modules were significantly negatively correlated with TG content (r=-0.5, p = 0.005; r=-0.51, p = 0.04;r=-0.46,p = 0.01;-0.71,p = 1e-5;r=-0.64,p = 2e-04), and the brown, lightgreen, and grey60 modules were significantly positively correlated with TG content (r = 0.71, P = 1e-0.5; r = 0.37, p = 0.04;r = 0.45, p = 0.01;), with the brown module showing the strongest correlation with TG content. Seven modules, namely the brown, pink, lightcyan, turquoise, green, black and cyan modules, were significantly correlated with PLIP content (P < 0.05), among which the pink, lightcyan, cyan, green, black, and turquoise modules were significantly negatively correlated with PLIP content (r=-0.63, p = 2e-04; r=-0.39,p = 0.03;r=-0.4,p = 0.03, r=-0.51, p = 0.04;r=-0.68,p = 3e-05;r=-0.79,p = 2e-07), and brown was significantly positively correlated with PLIP content (r = 0.84, p = 9e-09), showing the strongest correlation.

### Selection of candidate genes in relevant modules

Database After annotating the genes in the relevant modules using the BioMart database, we selted genes associated with fat deposition based on their functional annotation. There were 134 candidate genes associated with fat development in TG-related modules (Table 4), and 145 candidate genes associated with fat development in PLIP-related modules (Table 5). According to the above results, this experiment identified candidate genes that affect fat deposition and development.

**Table 4:** Candidate genes in TG-related modules

Module Color	Candidate genes
brown	*SIX1, NAPEPLD, ADIPOR1, ZC3H12A, PLA2G7, ACBD5, DAGLA, ABCA2.etal*
pink	*FGF10, E2F1, BBS4, BRCA1, PRIMPOL, CDK1, GJC2, SARDH, SOX8.etal*
turquoise	*PID1, ACADL, PANK2, AGTR1, ACBD7, HACD2, PTGS1, CD36, VDAC2.etal*
green	*ID4, PPARGC1A, DPEP1, SLC27A4, SGPL1, ACSL1, PLA2G15, GPCPD1*,
black	*FNDC5, ACAD9, PLCXD3, TIMM44, PPARA, C1QBP, ACAD8, MARS2.etal*

**Table 5:** Candidate genes in PLIP-related modules

Module Color	Candidate genes
brown	*SIX1, NAPEPLD, ADIPOR1, NFATC1, PPP3CA, PPP3CC, MED1.etal*
pink	*FGF10, E2F1, BBS4, BRCA1, PRIMPOL, CDK1, GJC2, SARDH, SOX8.etal*
turquoise	*METRNL, CCND1, PID1, TMEM120A, H-RAS, RNASEL, TCF7L2.etal*
green	*ID4, PPARGC1A, DPEP1, SLC27A4, SGPL1, ACSL1, PLA2G15.etal*
black	*FNDC5, ACAD9, PLCXD3, TIMM44, ECI2, NDUFAB1, FABP3.etal*

### Hub genes in fat deposition-related modules

**Fig. 4:**
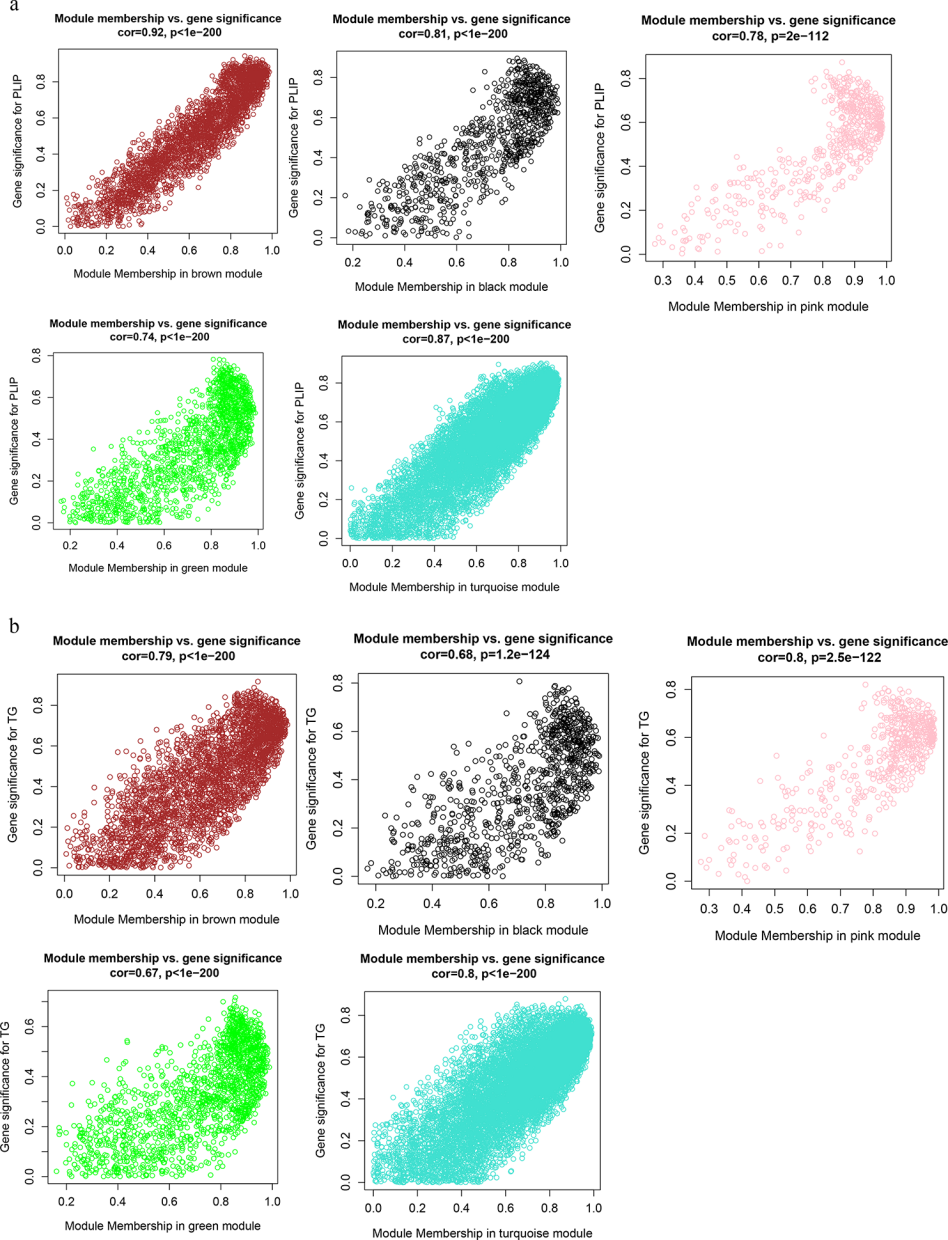
Scatter plot of the GS and MM. **(a)** Scatter plot of the GS and MM correlation in TG related modules. **(b)** Scatter plot of the GS and MM correlation in PLIP related modules. The ordinate axis represents the correlation between gene and phenotype, the abscissa axis represents the correlation between gene and module, and different colors represent different modules. The gene in the upper right corner of each figure is highly phenotype-related and is the hub gene in the module

**Fig. 5:**
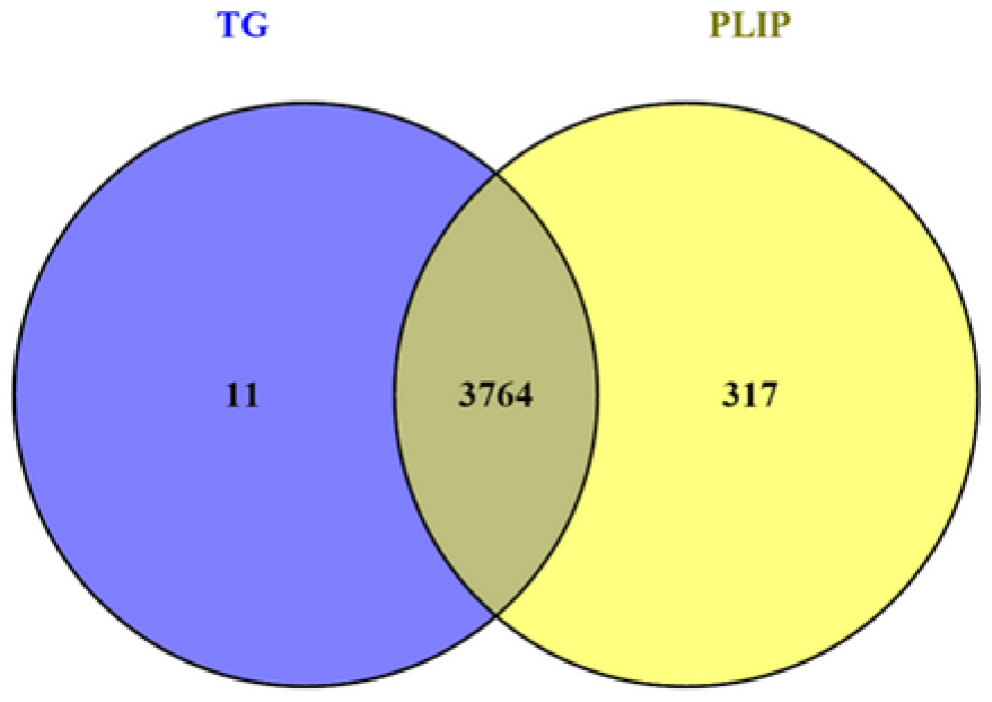
Overlap of hub genes in the TG and PLIP-related modules, overlapping hub genes are important genes

**Fig. 6:**
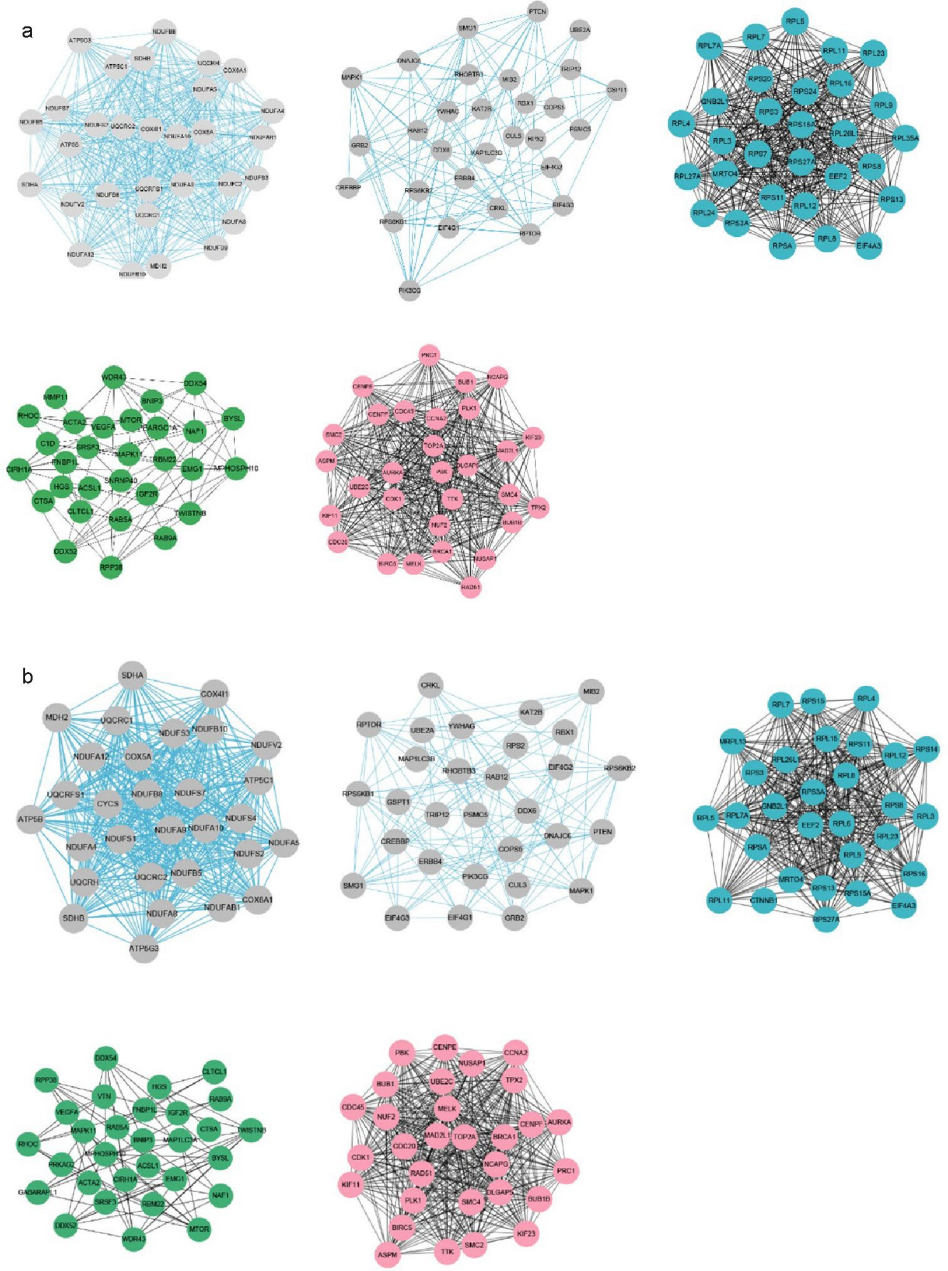
Visualization of the top 30 genes in specific modules: black, brown, turquoise, green, pink. **(a)** Top 30 hub genes with the highest degree of connectivity in TG-related modules. **(b)** Top 30 hub genes with the highest degree of connectivity in PLIP-related modules. The thicker the line, the higher the correlation, and more lines indicate that the gene is more important

A module that is significantly associated with the IMF-related phenotype means that the genes in the module are involved and play a key role in IMF formation. Therefore, for follow-up analysis, we focused on the genes in the module. To identify the hub genes in the module, the scatter plot of GS and MM correlations in the module was drawn (Fig. 4a, b), and the genes with |GS|>0.2 and |MM|>0.8 from each of the 5 modules associated with TG and PLIP were selected as the hub genes. We found 865, 390, 1539, 612, and 369 hub genes significantly associated with TG in the brown, pink, turquoise, green and black modules, respectively. We also found 872, 387, 1662, 684 and 473 hub genes significantly associated with PLIP in the brown, pink, turquoise, green and black modules, respectively. Overlapping hub genes identified by association with TG and hub gene identified by association with PLIP revealed that most of the genes overlapped (Fig. 5). Then, after constructing the gene co-expression network using Cystoscope3.9.0, the top thirty genes with the highest degree of connectivity in each module were selected as the true hub genes. Ultimately, 150 hub genes associated with TG and 150 hub genes associated with PLIP were identified, and the identified hub genes were visualized with Cytoscape 3.9.0 (Fig. 6a, b).

### Functional enrichment analysis of TG and PLIP-related hub genes

**Fig. 7:**
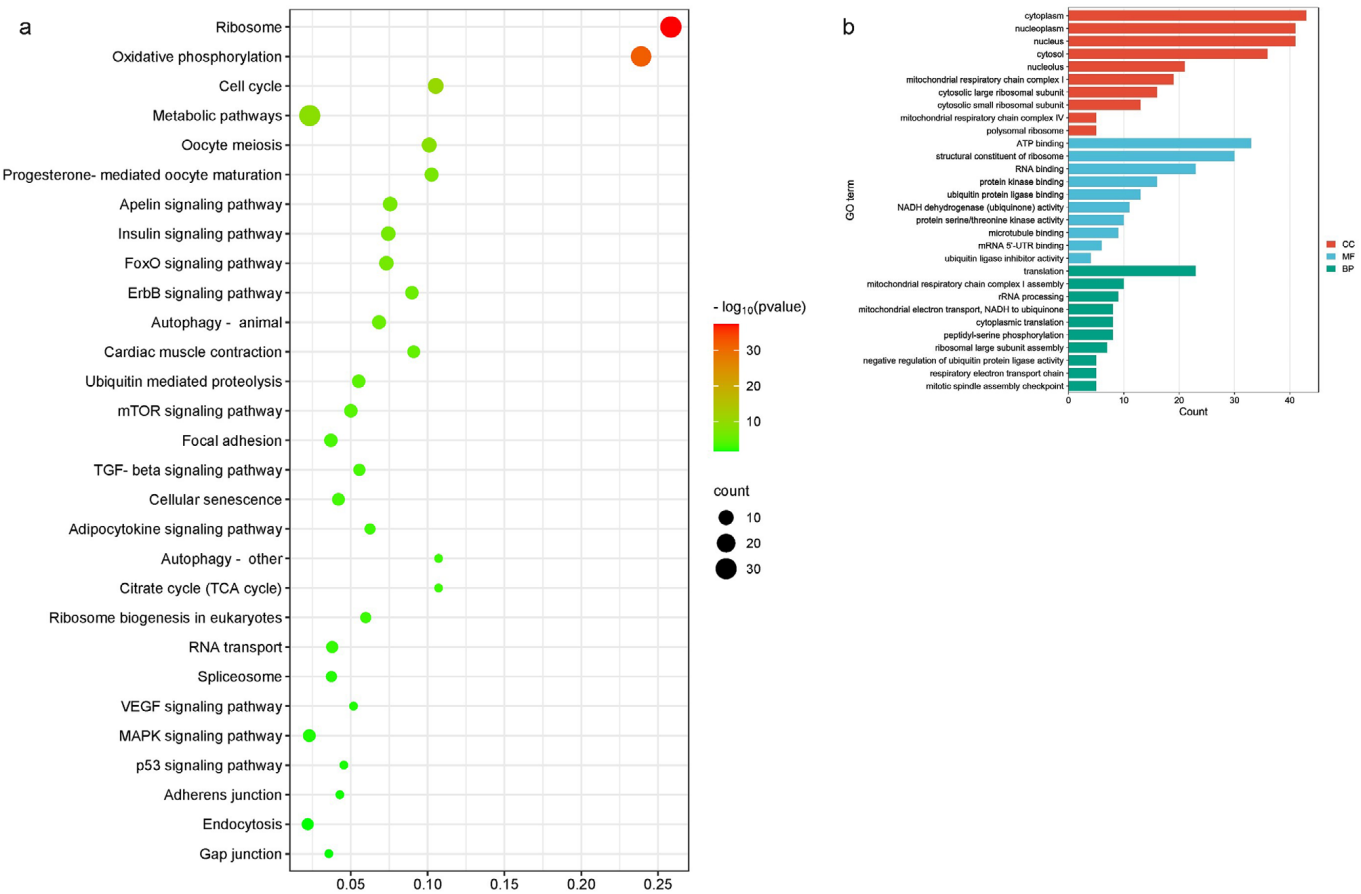
Pathway enrichment analysis **(a)** and GO term annotation **(b)** of genes in the modules. BP, biological processes; CC, cellular components; MF, molecular functions

**Fig. 8:**
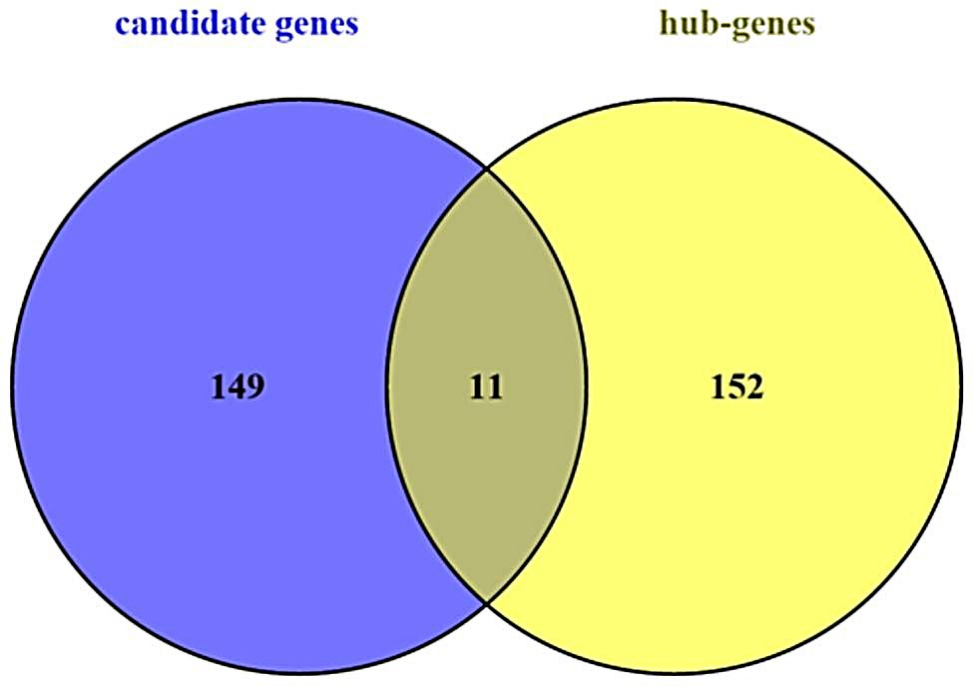
According to the Wayne diagram of correlation analysis, overlapping genes are important candidate genes. Intersection of hub gene and candidate gene in TG and PLIP related module;

The KEGG enrichment analysis of hub genes in the modules was mainly focused on important signaling pathways, such as the adipocytokine signaling pathway, MAPK signaling pathway, mTOR signaling pathway, FoxO signaling pathway, ErbB signaling pathway, TGF-beta signaling pathway, etc. GO term annotations were used to select the top ten enriched most significant terms in BP, CC, and MF (Fig. 7a, b). These GO terms were mainly annotated to translation (GO:0006412), mitochondrial respiratory chain complex I (GO:0005747) and structural constituent of ribosome (GO:0003735). The BPs annotated to these GO terms are all related to the formation of lipids. Among the 150 TG-related candidate genes nine hub genes, namely *RPS6KB1, BRCA1, CDK1, RPS3, PPARGC1A, ACSL1, NDUFAB1, NDUFA9 and ATP5B*, are also hub genes in the TG-related module, and among the 150 PLIP-related candidate genes, *NDUFAB1, NDUFA9, ATP5B, PRKAG2, ACSL1, BRCA1, CDK1 and RPS3* are the hub gene in the PLIP-related module. Therefore, *RPS6KB1, BRCA1, CDK1, RPS3, PPARGC1A, ACSL1, NDUFAB1, NDUFA9, ATP5B and PRKAG2* may be the key genes affecting IMF deposition(Fig. 8). These genes are mainly enriched in Adipocytokine signaling pathway, Insulin signaling pathway, FoxO signaling pathway.

## Discussion

At present, there are much research on intramuscular fat in poultry at home and abroad, and important progress has been made. For example, the main signaling pathways and genes involved in the regulation of intramuscular fat during development were explored; The molecular mechanism of fat differential deposition regulation was revealed by studying the difference of fat in different parts, and the biochemical mechanism of IMF influence on meat flavor was also analyzed. In general, the existing research mainly focus on the screening of candidate genes and pathways of IMF, and the deep-seated molecular mechanisms of its directed deposition regulation remain to be studied.

IMF is a lipid mixture composed of TG, PLIP and TCHO closely related to meat quality [26], of which TG and PLIP are the most abundant. TG, as the main component of IMF, is closely related to fat metabolism and is a representative indicator of IMF content [27]. Therefore, increasing the content of TG is an important way to regulate IMF content, while the synthesis of TG and PLIP has a crucial role in fat deposition in muscles. It is well established that IMF deposition is a complex physiological process mediated through various mechanisms involving multiple metabolic and signaling pathways regulated by proteins encoded by many functional candidate genes Several studies have shown that SLC16A7 is involved in TG deposition and mainly plays a role in muscle cells [28].The developmental stage is the major muscle and IMF formation period where myocytes and adipocytes interact when they are in close proximity in muscle tissue [29]. Thus, the identification of co-expressed genes in developing muscle tissue is important to understand the muscle formation process and the regulation of meat quality, which can reveal the molecular regulatory relationships between them. In this study, samples of JXY chicken breast muscle were collected from seven different developmental stages, namely D1 (first day after birth), D7, D35, D63, D91 and D119, and subjected to transcriptome sequencing and WGCNA to identify genes associated with IMF deposition at different developmental stages.

WGCNA can make full use of phenotypic information, convert the association between many genes and phenotypes into the relationship between gene set and phenotype, and cluster genes with similar expression patterns into one module, therefore we use WGCNA to construct a gene co-expression network [30]. at different developmental stages and identify key genes affecting IMF deposition according to TG and PLIP content phenotypes. WGCNA was used to identified 27 co-expression modules and found 8 and 7 modules significantly related to TG and PLIP, respectively. Additionally, it was also used to analyze the hub genes in the TG and PLIP significantly related modules and found that most of the genes affecting TG and PLIP overlapped. Since TG is composed of glycerol and fatty acids [31] and PLIP is composed of phosphate and fatty acids [32], we suggest that these genes affect the contents of TG and PLIP and thus IMF deposition mainly by affecting fatty acid content. Fatty acids are the basic components of cells [33], and their content and type are important factors affecting the nutritional value and taste of chicken meat as well as important precursors of volatile compounds [34], which play a key role in various biological processes, such as energy storage, metabolic regulation, and information transfer in animal bodies. Genetics and diet affect the composition of fatty acids, different fatty acids have different melting points, so for pigs and ruminants, fatty acids affect the hardness of adipose tissue [35].

Studies have shown that the *FABP* gene family is an important candidate gene family in the regulation of the IMF content, meat tenderness and flavor, and is related to lipid metabolism pathways, such as those involved in fat decomposition and production [36]. As a marker of adipocyte differentiation, *FABP4* can regulate the expression of *PPARG* and play an important role in fatty acid transport and metabolism [37].

Enrichment analysis of genes in TG- and PL-related modules found 39 significant signaling pathways, including some important signaling pathways, such as MAPK signaling pathway, mTOR signaling pathway, and FoxO signaling pathway. Some studies have shown that the MAPK signaling pathway can affect the production of fat and regulate the lipid metabolism through the PPAR signaling pathway [38]. Also, as a target gene of microRNA miR-29a, CTRP6 can promote the proliferation and differentiation of intramuscular and subcutaneous adipocytes through the MAPK signaling pathway [39].

In this study, candidate genes related to fat deposition in TG- and PL-related modules were identified. Specifically, 134 genes related to fat deposition in TG-related modules and 145 genes related to fat deposition in PLIP-related modules were identified. Among the top 30 selected hub genes with the highest connectivity in TG- and PL-related modules, *RPS6KB1, BRCA1, CDK1, RPS3, PPARGC1A, ACSL1, NDUFAB1, NDUFA9* and *ATP5B* genes were found to be both candidate genes related to adipogenesis and highly linked hub genes in the TG-related module, while *NDUFAB1, NDUFA9, ATP5B, PRKAG2, ACSL1, BRCA1, CDK1* and *RPS3* were found to be both adipogenesis-related and highly linked hub genes in the PLIP-related module. Therefore, *RPS6KB1, BRCA1, CDK1, RPS3, PPARGC1A, ACSL1, NDUFAB1, NDUFA9, ATP5B and PRKAG2* may be key genes affecting IMF deposition in chickens.

Most of the genes are related to fat deposition, some studies have found that the *ELOVL6* gene is positively correlated with the proliferation of bovine precursor adipocytes, and the knockdown of *ELOVL6* expression significantly downregulated the protein expression levels of *CDK1* and *PCNA*, prolonging the cell cycle transition from G2 phase to M phase, thus inhibiting the proliferation of precursor adipocytes [40]. Another study found that stearic acid is transported to dairy cow mammary epithelial cells through *FATP4*, upregulating the expression of *CDK1*, and then activating the PI3K-mTOR-4EBP1/S6K and mTOR-SREBP-1 signaling axes to promote the synthesis of milk fat milk protein [41]. Research indicated a remarkable upregulation of *BRCA1* in the LW pig, signifying its pivotal role in modulating fatty acid and lipid metabolism, alongside controlling obesity [42]. Likewise, *PPARGC1A*, acting as a transcription factor, exerts profound influence over the regulation of white adipocyte differentiation and the deposition of bovine intramuscular fat (IMF) [43]. Furthermore, research has shown *PPARGC1A* expedites the accumulation of IMF through its positive regulation of saturated and monounsaturated fatty acid metabolism [44].

Investigations have demonstrated that the overexpression of *NDUFAB1* confers substantial protection against obesity and insulin resistance in murine models [45]. The porcine *ATP5B* gene exhibits extensive transcriptional expression across all tissues, with particular prominence in the delicate testis and adipose tissue, thereby implicating its involvement in skeletal muscle development and meat quality [46]. Moreover, *PRKAG1*, a distinguished member of the AMPK (AMP-activated protein kinase) family, substantially influences pork quality and backfat thickness [47]. Nevertheless, current literature remains scant in regard to the exploration of *RPS6KB1, RPS3, CDK1*, and *NDUFA9* genes in adipose tissue. Thus, further investigations are imperative to unravel the intricate associations between the proteins, encoded by these genes, and their involvement in fat deposition.

## Conclusion

Ten modules with significant correlation with IMF traits were identified by WGCNA. *RPS6KB1, BRCA1, CDK1, RPS3, PPARGC1A, ACSL1, NDUFAB1, NDUFA9, ATP5B* and *PRKAG2* are not only candidate genes related to fat development but also hub genes in related modules, which may be important candidate genes regulating IMF deposition in chickens. Hub genes in WGCNA module mainly affect TG and PLIP contents by regulating fatty acid metabolism, thereby affecting IMF deposition. It is of great significance to improve meat quality and meat safety to meet people’s demand for high-quality ecological products and improving breeding technology.

## Data Availability

The RNA sequencing raw data reported in this paper have been deposited in the Genome Sequence Archive (Genomics, Proteomics & Bioinformatics 2021) in National Genomics Data Center (Nucleic Acids Res 2022), China National Center for Bioinformation / Beijing Institute of Genomics, Chinese Academy of Sciences (GSA: CRA011927) that are publicly accessible at https://ngdc.cncb.ac.cn/gsa.

## Declarations

This study was conducted in accordance with the Guide for the Care and Use of Laboratory Animals published by the Ministry of Science and Technology (Beijing, China). The study was approved by the Research Ethics Committee of the Institute of Animal Sciences of the Chinese Academy of Agricultural Sciences (Beijing, China) (Protocol No. IAS2019-21). All methods were carried out in accordance with relevant guidelines and regulations. This study was carried out in compliance with the ARRIVE guidelines (Animal Research: Reporting of In Vivo Experiments).

### Author details

^1^College of Animal Science and Technology, Qingdao Agricultural University, Qingdao 266109, China.

^2^State Key Laboratory of Animal Nutrition, Key Laboratory of Animal (Poultry) Genetics Breeding and Reproduction, Ministry of Agriculture, Institute of Animal Sciences, Chinese Academy of Agricultural Sciences, Beijing 100193, China.

^3^Guangxi Jingling Agriculture and animal Husbandry Group Co., LTD,Nanning 530049,China.
